# Supplementary material for: Conformational States of the GDP- and GTP-Bound HRAS Affected by A59E and K117R: An Exploration from Gaussian Accelerated Molecular Dynamics
Source: Molecules. 2024 Jan 30;29(3):645. doi: 10.3390/molecules29030645 (PMC10856033; doi:10.3390/molecules29030645)
Supplement: Supplementary file 1 [file molecules-29-00645-s001.zip › molecules-2767968-supplementary.pdf]

Conformational States of the GDP- and GTP-Bound HRAS Affected by A59E and K117R: An Exploration from Gaussian Accelerated Molecular Dynamics

Zhiping Yu [1], Zhen Wang [2], Xiuzhen Cui [2], Zanxia Cao [1], Wanyunfei Zhang [3], Kunxiao Sun [3] and Guodong Hu [1,*]

[1] Shandong Key Laboratory of Biophysics, Dezhou University, Dezhou 253023, China;

[2] Pingyin People's Hospital, Jinan 250400, China

[3] School of Science, Xi'an Polytechnic University, Xi'an 710048, China

*Correspondence: xzszhgd@163.com

File S1. The details of GaMD reweighting

The free energy can be reweighted through the reweighting program developed by Miao et al. The reaction coordinates (RCs) are divided into different bins at a certain interval. The probability distribution of selected reaction coordinates can be calculated from the GaMD trajectory as $p^*(A)$. Given the boost potential ΔV(r) of each conformation recorded in the GaMD trajectory, $p^*(A)$ can be reweighted to detect the canonical ensemble distribution, $p(A)$, based on the following equation:

$$\mathrm{p}(A_j) = p^*(A_j)\frac{<e^{\beta\Delta V(r)}>_j}{\sum_{i=1}^{M}<p^*(A_i)e^{\beta\Delta V(r)}>_i} \qquad j = 1, \ldots, M$$

in which M is the number of bins, $\beta = k_B T\,\beta$, and $< e^{\beta\Delta V(r)} >_j$ is the ensemble-averaged Boltzmann factor of $\Delta V(r)$ for simulation conformations detected in the *j*th bin. The ensemble-averaged reweighting factor can be approximated using cumulant expansion [1-3]

$$< e^{\beta\Delta V(r)} = exp\left\{\sum_{k=1}^{\infty}\frac{\beta^k}{k!}C_k\right\}$$

where the first two cumulants are determined based on the following equation

$$C_1 =< \Delta V >,$$

$$C_2 =< \Delta V^2 > -< \Delta V >^2 = \sigma_V^2$$

In general, the boost potential resulting from GaMD simulations has a near-Gaussian distribution [4]. As a result, cumulant expansion to the second order provides a good approximation for computing the reweighting factor. The reweighted free energy $F(A) = -k_B T ln(\rho_A)$ is obtained from the following equation

$$F(\mathrm{A}) = F^*(A) - \sum_{k=1}^{2}\frac{\beta^k}{k!}C_k + F_C$$

in which $F^*(A) = -k_B T ln p^*(A)$ is the modified free energy obtained from GaMD simulation, and F_C is a constant.

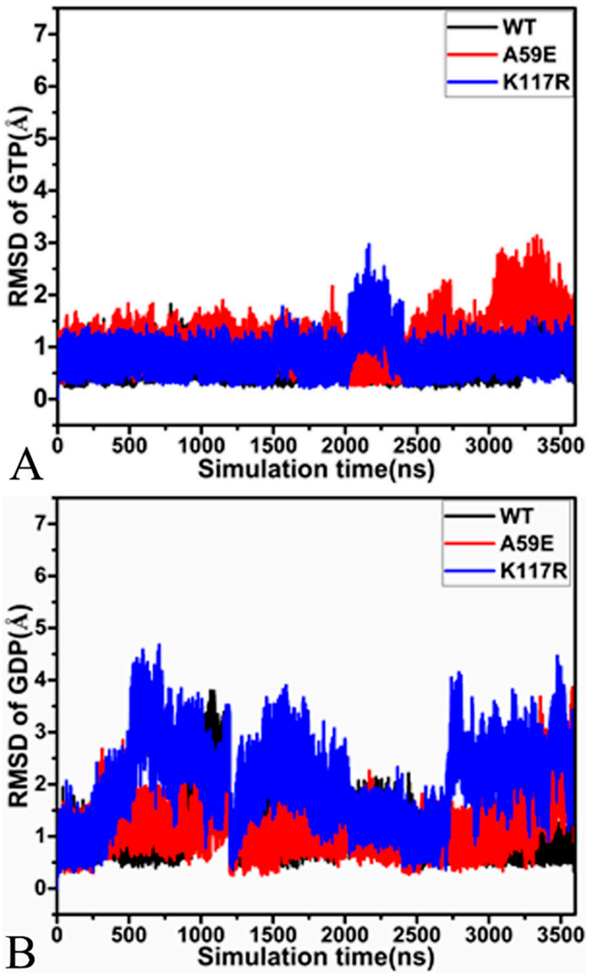

Figure S1. The function of RMSDs for heavy atoms from GTP and GDP as the simulation time.

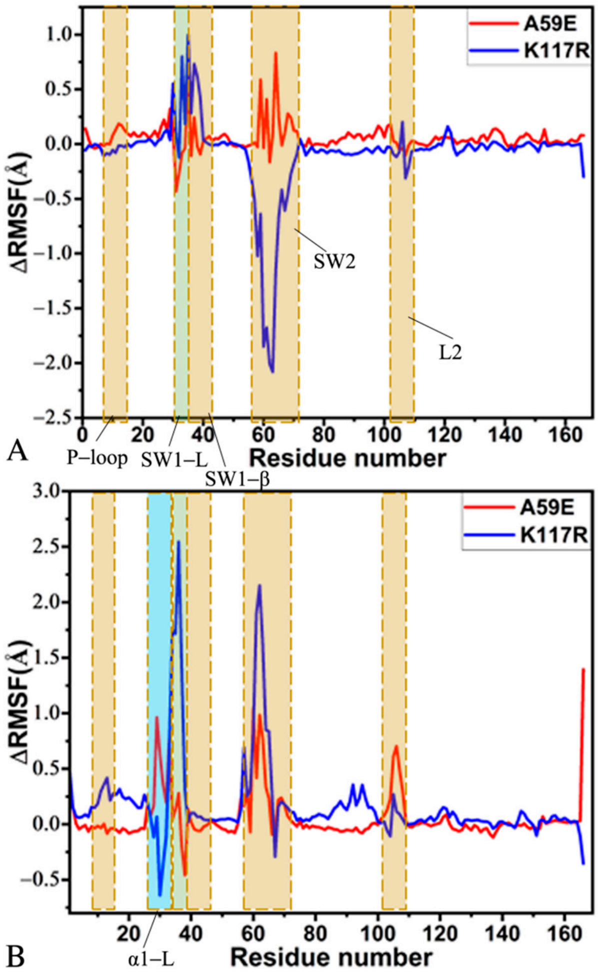

Figure S2. The difference of RMSFs between the GTP/GDP-bound mutants and the GTP/GDP-bound WT HRAS: (A) GTP and (B) GDP

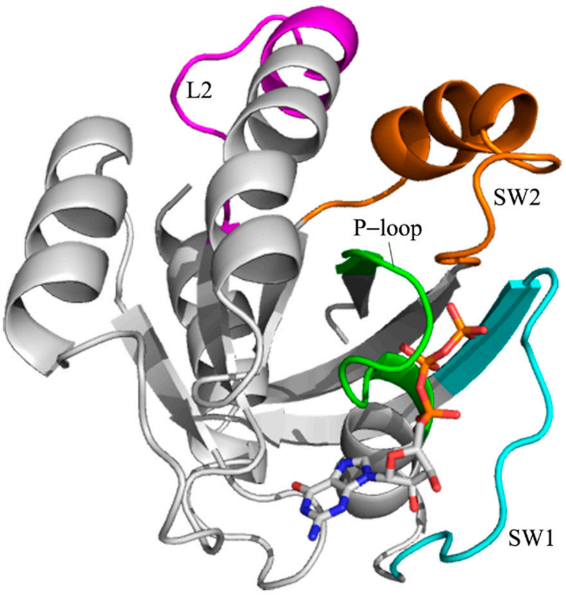

Figure S3. The structural domains corresponding to the obvious changes of the RMSFs. The HRAS is shown in cartoon modes and the ligand is displayed in stick modes.

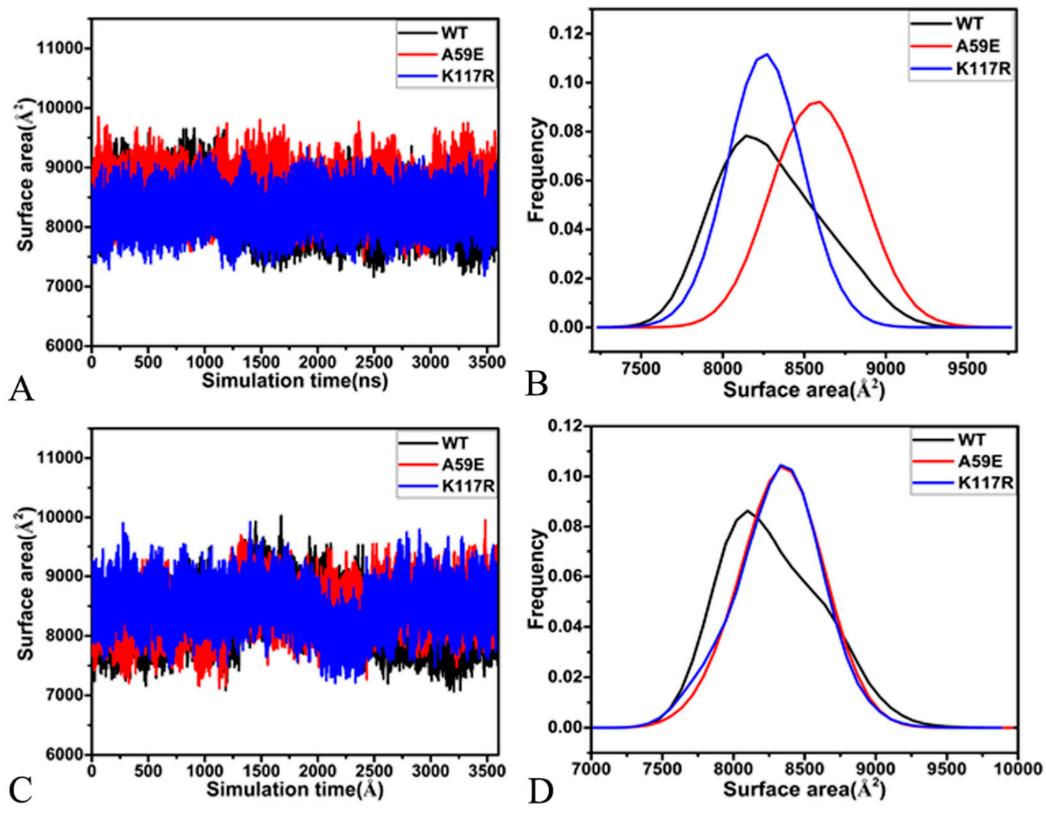

Figure S4. MSAs of the GTP- and GDP-bound HRAS: (A) and (B) corresponding to the time course and frequency distribution of MSAs for the GTP-bound WT, A59E and K117R HRAS, respectively, and (C) and (D) corresponding to the time course and frequency distribution of MSAs for the GDP-bound WT, A59E and K117R HRAS.

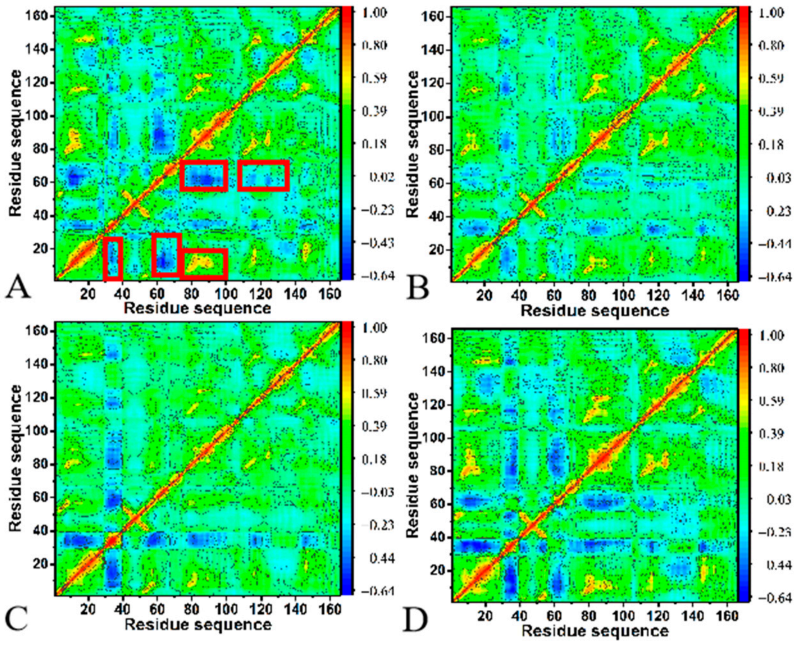

Figure S5. DCCMs calculated by using the coordinates of the Cα atoms in HRAS: (A) and (C) corresponding to the DCCMs of the GTP-bound A59E and K117R HRAS, respectively, and (B), and (D) corresponding to the DCCMs of the GDP-bound A59E and K117R HRAS, individually.

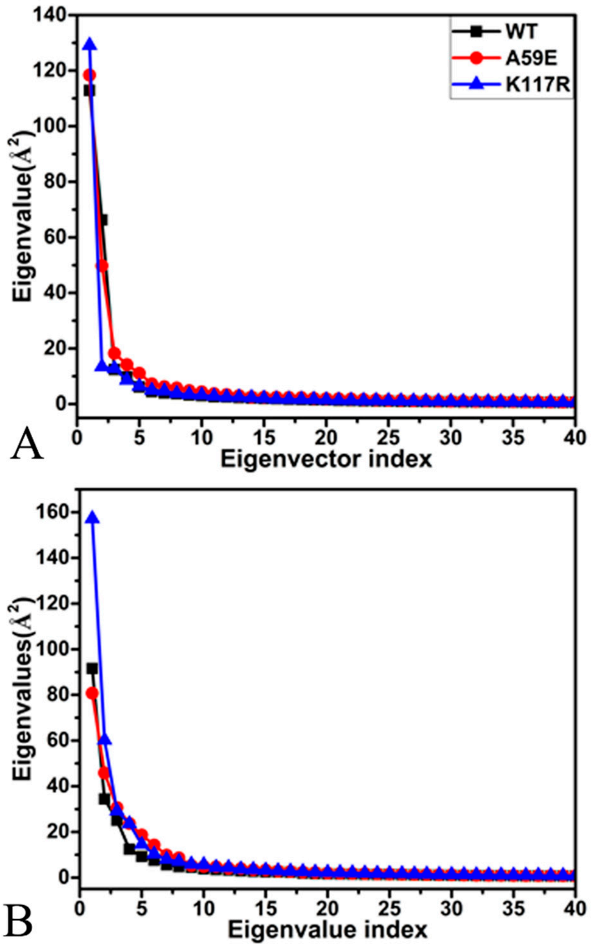

Figure S6. The function of eigenvalues arising from PCA as the eigenvector indexes: (A) the GTP-bound WT, A59E and K117R HRAS and (B) the GDP-bound WT HRAS.

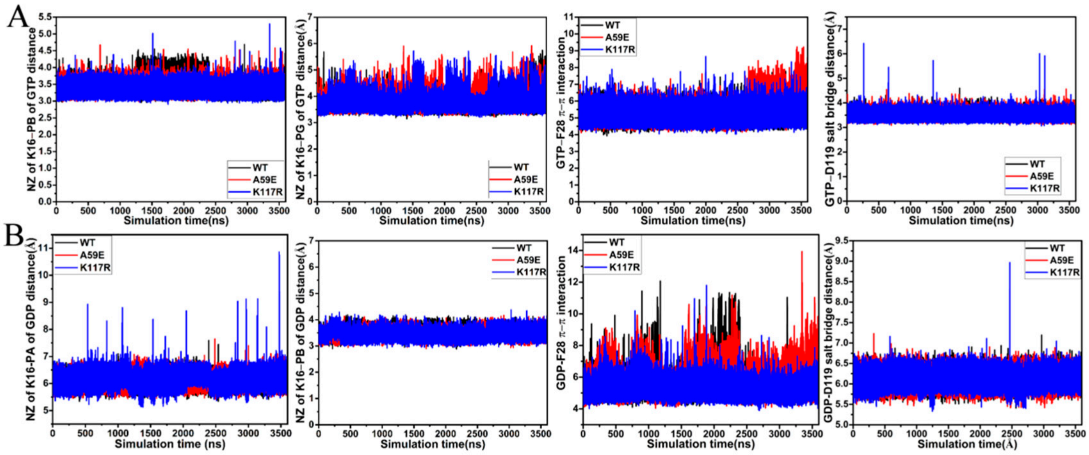

Figure S7. Time course of the distances for GTP-residue (A) and GDP-residue interactions (B).

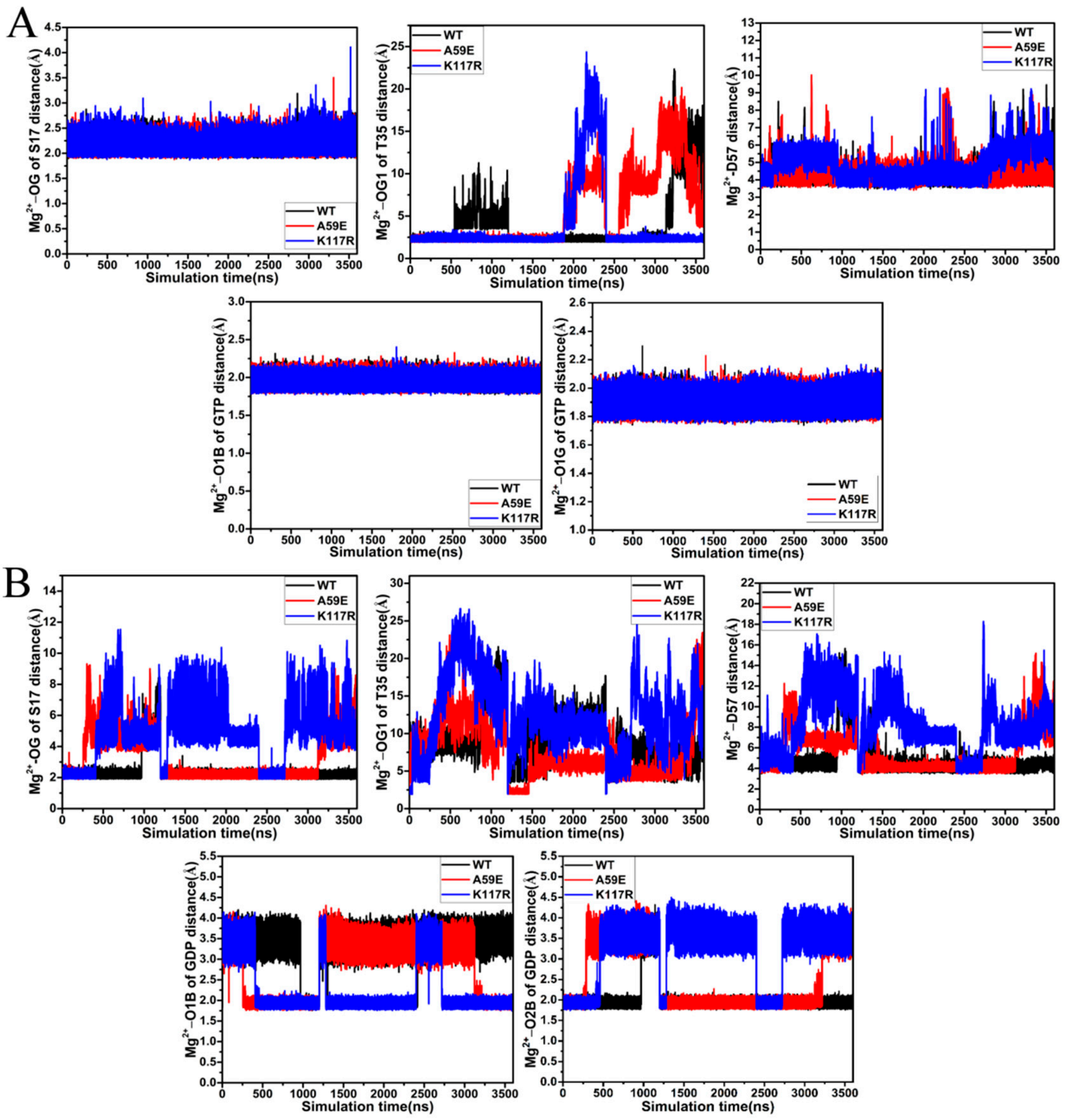

Figure S8. Time course of the distances involved in the Mg^{2+} interactions in the GTP-bound (A) and GDP-bound HRAS systems (B).

References

1. Miao, Y.; Feher, V. A.; McCammon, J. A. Gaussian Accelerated Molecular Dynamics: Unconstrained Enhanced Sampling and Free Energy Calculation. *J. Chem. Theory Comput.* **2015**, *11*, 3584-3595.
2. Miao, Y.; Sinko, W.; Pierce, L.; Bucher, D.; Walker, R. C.; McCammon, J. A. Improved Reweighting of Accelerated Molecular Dynamics Simulations for Free Energy Calculation. *J. Chem. Theory Comput.* **2014**, *10*, 2677-2689.
3. Do, H. N.; Wang, J.; Miao, Y. Deep Learning Dynamic Allostery of G-Protein-Coupled Receptors. *JACS Au* **2023**, *3*, 3165-3180.
4. Miao, Y.; McCammon, Implementation and Applications. J. A. *Annu. Rep. Comput. Chem.* V13 231-278.
